# A decision-analytic approach to predict state regulation of hydraulic fracturing

**DOI:** 10.1186/s12302-014-0020-7

**Published:** 2014-08-02

**Authors:** Igor Linkov, Benjamin Trump, David Jin, Marcin Mazurczak, Miranda Schreurs

**Affiliations:** 1 US Army Engineer Research and Development Center, 696 Virginia Rd, Concord, MA 01742 USA; 2 Free University of Berlin, Kaiserswerther Straße 16-18, Berlin, 14195 Germany; 3 Wrocław University of Technology, Wybrzeże Stanisława Wyspiańskiego 27, Wrocław, 50-370 Poland; 4 Massachusetts Institute of Technology, 77 Massachusetts Avenue, Cambridge, MA 02139 USA

**Keywords:** Hydraulic fracturing, Multi criteria decision analysis, Policy alternatives, Energy policy

## Abstract

**Background:**

The development of horizontal drilling and hydraulic fracturing methods has dramatically increased the potential for the extraction of previously unrecoverable natural gas. Nonetheless, the potential risks and hazards associated with such technologies are not without controversy and are compounded by frequently changing information and an uncertain landscape of international politics and laws. Where each nation has its own energy policies and laws, predicting how a state with natural gas reserves that require hydraulic fracturing will regulate the industry is of paramount importance for potential developers and extractors. We present a method for predicting hydraulic fracturing decisions using multiple-criteria decision analysis. The case study evaluates the decisions of five hypothetical countries with differing political, social, environmental, and economic priorities, choosing among four policy alternatives: open hydraulic fracturing, limited hydraulic fracturing, completely banned hydraulic fracturing, and a cap and trade program.

**Results:**

The result is a model that identifies the preferred policy alternative for each archetypal country and demonstrates the sensitivity the decision to particular metrics. Armed with such information, observers can predict each country’s likely decisions related to natural gas exploration as more data become available or political situations change.

**Conclusions:**

Decision analysis provides a method to manage uncertainty and address forecasting concerns where rich and objective data may be lacking. For the case of hydraulic fracturing, the various political pressures and extreme uncertainty regarding the technology’s risks and benefits serve as a prime platform to demonstrate how decision analysis can be used to predict future behaviors.

## Background

Horizontal drilling and high-volume hydraulic fracturing, collectively known as ‘fracking,’ opened up the possibility for new natural gas extraction across the globe. While not a novel technology, fracking has taken off in the USA and Canada in regions with substantial yet traditionally difficult to harvest shale gas [1]. Despite the potential for economic benefits, the use of such technology introduces risks to humans and the environment [2]. These risks are compounded over the extended time period by which wells tapping rich natural gas deposits remain in operation [3]. Uncertainty regarding the likelihood and consequences of harmful events has driven many local, regional, and national governments to issue warnings, regulations, and moratoriums on the industry [4,5]. Natural gas companies are challenged to identify rich deposits that can be extracted as cheaply and efficiently as possible with the longest expected payoff, while at the same time hedging against the likelihood that industry regulations will change. As such, the ability of a company to predict future state behavior in regulating a highly uncertain practice is critical to its long-term survival and success.

In this paper, we present a decision tool that simulates state behavior with regard to the regulation of hydraulic fracturing. This tool takes into account a variety of factors ranging from drill site profitability to the public perception of hydraulic fracturing as an acceptable practice, a realistic enumeration of the various pressures facing government legislators currently considering how to regulate the industry. We make particular use of decision analysis to aggregate a variety of quantitative and qualitative state inputs, which is ultimately used to produce a prediction regarding state action towards hydraulic fracturing. While this demonstration is a fictional representation of state action by making use of archetypal countries and hypothetical data, users could input their own data to construct a more realistic set of predictions for a chosen set of states.

## Results and discussion

**Figure 1:**
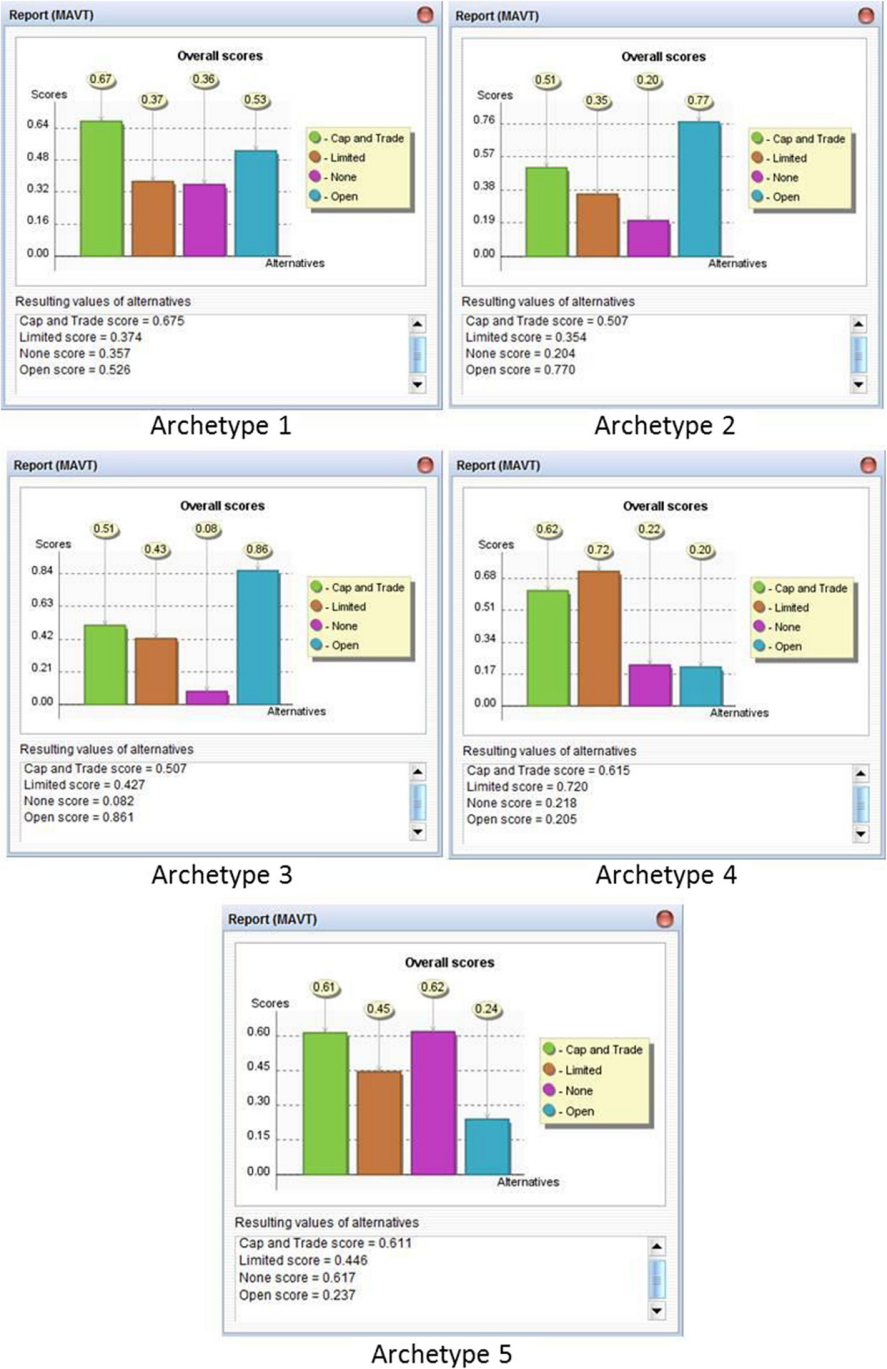
**MCDA policy alternative scores.**

The overall scores displayed in Figure 1 represent the weighted summation of value functions applied to each alternative for each archetype. The main result is a ranked list of policy alternatives for each archetypal country. The policy alternative with the highest score is the most preferred option, while the alternative with the lowest score is the least preferred option. Each archetypal country has a current optimal policy solution - although some are more resolute than others. The model output (Figure 1) for each country indicates a ranking of the policy options based upon their relative optimality, indicating where a policy option is clearly favored (e.g., open hydraulic fracturing in country 3), or where results are less clear and require a more thorough sensitivity analysis, such as with country 5. Ultimately, each country’s policy options serve as a mathematical reflection of the political, environmental, economic, and social pressures facing national lawmakers. For example, within country 1 (developed capitalist democracy), rich gas reserves coupled with strong fuel consumption reduce preference for the option of banning hydraulic fracturing altogether, yet existing environmental concerns (having the highest criterion weight) also reduce preference for only limited regulation. Consequently, a cap and trade system for gas well development rises as a politically and economically acceptable alternative.

**Figure 2:**
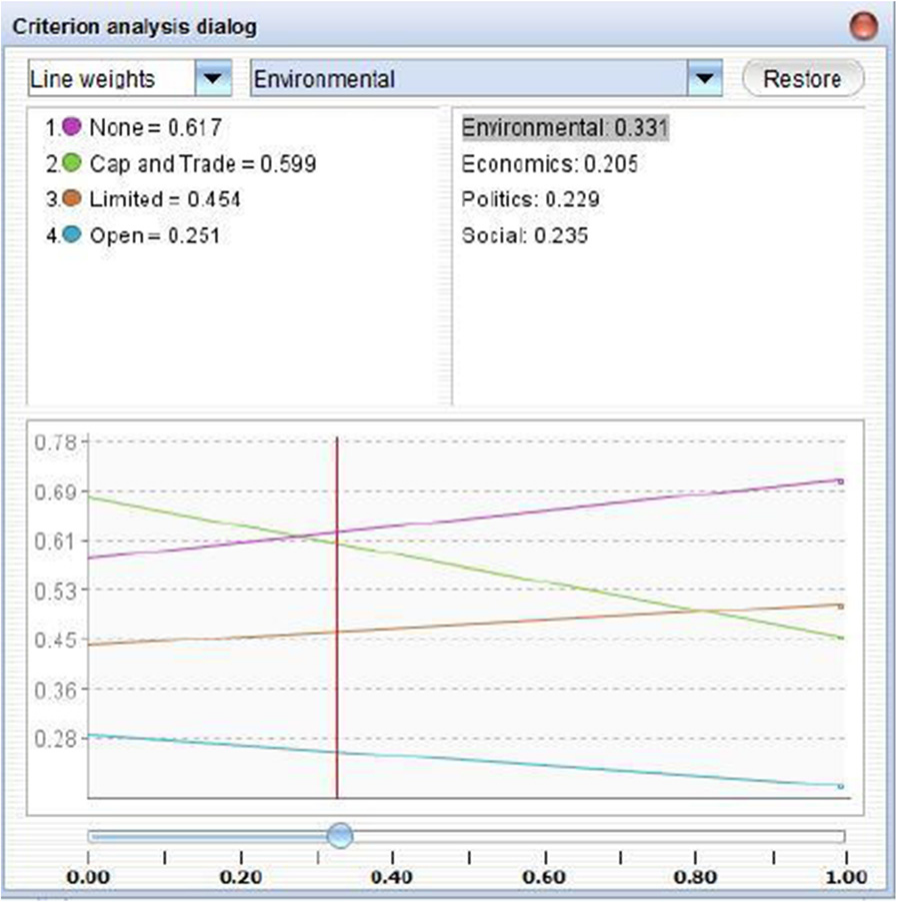
**Sensitivity analysis for weight of Environment criterion for archetype 5.**

**Figure 3:**
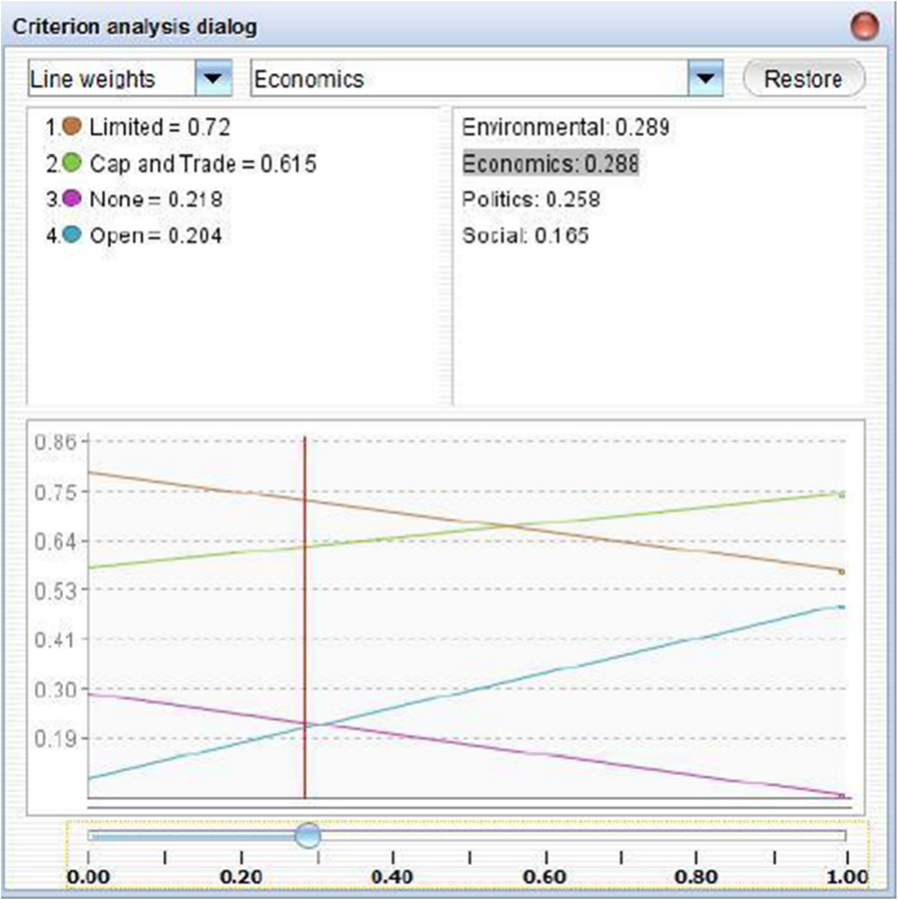
**Sensitivity analysis for weight of Economics criterion for archetype 4.**

Due to the strong tendency of preferences to shift within public policy, understanding how changes in the elements of the hydraulic fracturing decision model impact each country’s preferred policy alternative is critical to reducing uncertainty. Sensitivity analysis is helpful to analyze how a shift in a specific criterion or sub-criterion’s weight affects the overall decision. Figures 2 and 3 display the sensitivity analysis output for the ‘Environment’ criterion of archetype 5 and the ‘Economics’ criterion of archetype 4, respectively. The vertical axes show the value of each policy alternative as a function of the weight of the criterion by percent on the horizontal axes. Figure 2 shows the preference score for each alterative as a function of the weight applied to the Environment criterion in country 5. The intersections illustrate the critical points where the preferred alternative changes from the current policy alternative to a different one. Here, even a minor decline in the importance of Environment relative to the other criteria could contribute to a shift from ‘no hydraulic fracturing’ to ‘cap and trade’. As such, this weighting scheme is considered ‘sensitive’ and should be considered alongside any expected developments in national priorities. For archetype 4, the weight of the Economics criterion must increase from 29% to over 55% in order for the preferred policy alternative to transition from ‘limited’ to ‘cap and trade,’ indicating that this archetypal country is relatively insensitive to economic criteria. Such an analysis can be conducted on all decision criteria for all countries in order to anticipate future shifts in priorities and preferences and determine the point where a different policy option may become a better choice.

## Conclusions

With this hypothetical case study, we demonstrate how decision analysis can be used to predict the behavior of governments in anticipation of hydraulic fracturing policy. Such a tool could prove valuable not only to drilling companies whose livelihoods depend upon understanding and predicting drilling regulation but also to academics and researchers seeking to gain greater understanding and transparency of how different political pressures impact high-level decision making. This form of decision aid is particularly helpful to better understand the breadth of issues and concerns facing policymakers along with the uncertainty regarding how future developments may affect major policy decisions.

Though a skilled analyst is required to perform these computations, decision analysis (multi-criteria decision analysis (MCDA) in particular) can supplement traditional political science research in forecasting behavior on issues with subjective, disparate, or highly uncertain data. The archetypal examples above demonstrate how a variety of differing inputs may be aggregated and quantified in a manner where an analyst can meaningfully compare a host of policy options. In this case, organizations interested in hydraulic fracturing can use available information to predict to what degree where countries with gas deposits will regulate drilling. Such a decision tool could in turn reduce the uncertainty and confusion surrounding the variety of inputs to consider and guide organizational decision making over a span of time. Further sensitivity analysis can help describe how changes in available data or public sentiment might affect the preferred policy alternative of governing bodies. Such knowledge can help decision makers adapt quickly as new information becomes available.

Decision analysis is not perfect - it does rely upon expert elicitation to acquire traditionally qualitative information. However, it provides a method to manage uncertainty and address forecasting concerns where rich and objective data may be lacking. For the case of hydraulic fracturing, the various political pressures and extreme uncertainty regarding the technology’s risks and benefits serve as a prime platform to demonstrate how decision analysis can be used to predict future behaviors.

## Methods

Multi-criteria decision analysis (MCDA) is a method for decision structuring that permits the use of both quantitative and qualitative data sources with high uncertainty or subjectivity [6]. Specifically, MCDA helps aggregate the impact of various unrelated inputs into a ranked list of quantitative results in a transparent process [7]. One type of qualitative data is stakeholder options, which in this study includes subjective metrics of a country’s willingness to accept certain hydraulic fracturing risks in order to acquire the perceived economic benefits. The Decision Evaluation for Complex Risk Network Systems (DECERNS) software [6,8] is used to incorporate stakeholder opinions with available data on the risks of unconventional drilling methods to determine to what degree each factor influences a state’s behavior towards hydraulic fracturing.

The MCDA prediction model requires the construction of a value hierarchy, a tree that represents the major factors and policy solutions which influence a stakeholder’s decision on a given issue [9]. These factors include the main criteria which pressure lawmakers and policymakers to regulate in a certain fashion. The first branch of value hierarchy development identifies the overarching criteria, including ‘Political,’ ‘Environmental,’ ‘Social,’ and ‘Economic’ factors. Further refined, these factors are broken down into individual elements which each represent a specific factor lawmakers must consider when forming hydraulic fracturing policy. The list discussed below is not exhaustive and can be expanded upon to meet the needs of a real world scenario.

**Figure 4:**
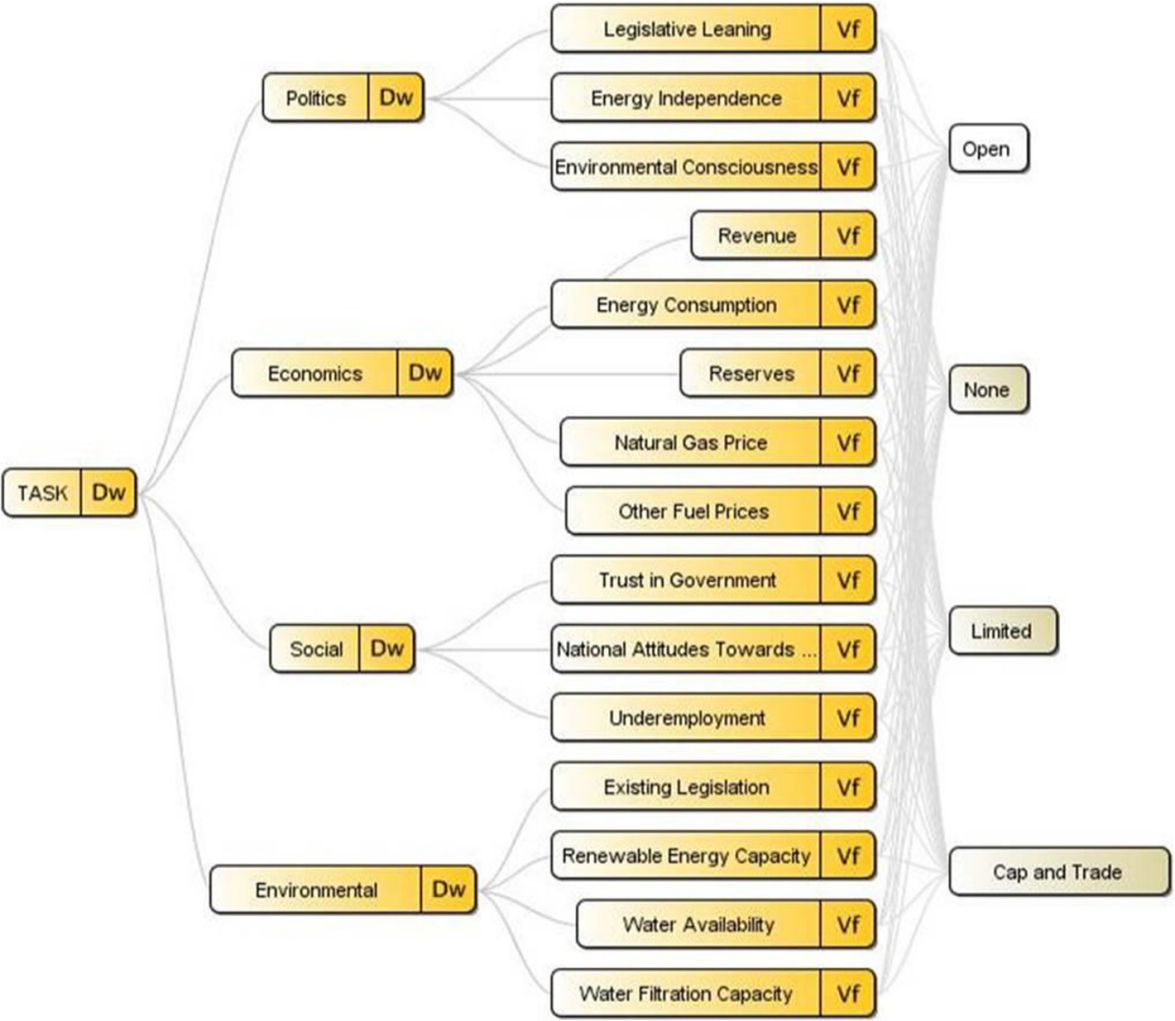
**Value hierarchy of the hydraulic fracturing state predictor.**

Political factors relate to the partisan behaviors of a state’s citizens and governing officials. State policy, influenced by public opinion, can shift quickly when the risks and benefits of hydraulic fracturing are relatively uncertain. One proposed element is the goal of *energy independence* (Figure 4), which considers the amount of energy that could be produced domestically, relative to current import levels, if hydraulic fracturing were to be officially sanctioned. Specifically, *energy independence* incorporates a state’s degree of desire to reduce reliance on foreign energy. Another element is *legislative leaning*, the proportion of a country’s legislature that is favorable towards hydraulic fracturing. *Legislative leaning* takes into account the general political affiliations of policymakers, under the belief that certain affiliations are prone to support or oppose hydraulic fracturing compared to others. A third example element for the Political factor includes *environmental consciousness*, which serves as a measure of the knowledge of environmental issues, especially those posed by hydraulic fracturing, of politicians and the general public.

Social factors include those that may have an effect upon a government’s ability and likelihood to regulate a potentially risky and uncertain activity such as hydraulic fracturing. Three elements - the degree of public *trust in government*, the *national attitudes towards hydraulic fracturing*, and the existing rate of *underemployment* within gas-rich regions - are used as measures of the public’s willingness to accept the risks of hydraulic fracturing in order to gain economic benefits (Figure 4). For example, it is assumed that the higher the level of underemployment within a gas-rich area, the more likely residents are to pressure their lawmakers to approve the further development of hydraulic fracturing sites.

Environmental factors focus explicitly upon the perceived risks that hydraulic fracturing activities pose to human and environmental health. As a more objective category, three elements that are analyzed here are the degree to which *existing environmental legislation* prevents/limits gas drilling, the *availability of other energy sources* (preferably renewable) within state borders, and the *availability of water* to be used in the hydraulic fracturing process (Figure 4). Of these factors, the availability of a relatively close and plentiful water supply is crucial to the drilling process, as millions of gallons of water are required by the fracturing technology. Without access to water, and the ability to safely dispose of it after use, unconventional drilling becomes strategically difficult to carry out or approve from a policymaking perspective.

Lastly, economic factors consider the direct benefits and potential financial limitations of hydraulic fracturing for a particular state. Benefits include an estimate of the expected revenue from hydraulic fracturing based upon the availability of gas resources and cost of compressing and transporting the extracted gas. Potential limitations to consider include the trend in prices for other energy sources, which will have a direct effect upon the demand and price for natural gas. Though not universally true, this factor will be a strong influence on most states’ decisions, as such natural gas serves as a method by which a state could procure millions of dollars in revenues that were previously unobtainable.

Ultimately, the development of the major decision criteria and their associated individual elements attempt to capture the pressures facing governmental officials required to act upon an industry where they have imperfect information. The value hierarchy for this policy problem is then expanded to include four potential policy outcomes: a full moratorium on hydraulic fracturing, a partial/regional/conditional moratorium on hydraulic fracturing, the limitation of drilling via a ‘cap and trade’ system, and a full and open allowance for all drilling (Figure 4). Other policy options certainly exist; however, for this case study, these options are chosen to show how the government officials’ decisions are further complicated by the presence of multiple policy outcomes. MCDA can be used to quantify the impact of each criterion on each of the policy alternatives. The quantified values serve as an approximate measure of the degree of pressure each sub-criterion expresses for lawmakers to regulate in a given way.

With the value hierarchy developed, a relative weighting scheme of the criteria would then be elicited from a selection of expert and stakeholder interviews. This qualitative information would be acquired using surveys or content analysis of such experts’ feedback, although for this case we utilize hypothetical weights. In general, weights are constructed in a manner where all branches of a criterion are normalized relative to their perceived importance. All major criteria (the four noted in this case) are additionally normalized across each other. Qualitative and quantitative scores are converted into a maximizing scale for each alternative. Mathematically, scores will aggregate ‘up the tree,’ where results will combine into a single risk attitude score for each individual alternative per country. For the final risk scores, higher scores correspond to less acceptance of risk (more negative risk attitude) regarding hydraulic fracturing. States with higher scores will see more restrictive policies regarding unconventional drilling as the preferred alternatives. The alternatives are subjectively ranked regarding their degree of regulatory restriction from least restrictive to most regulated as open, cap and trade, limited, and closed/no available drilling. Equation 1 gives a mathematical representation of the additive nature of the decision model:

1 $$ V(a)={w}_1\left({V}_1\left({a}_1\right)\right)+\dots +{w}_m\left({V}_m\left({a}_m\right)\right) $$

The alternatives tree is evaluated from the bottom up. At any particular level of interest, a value or utility function, *V*, is applied to the aggregate alternative scores, *a*, and is adjusted using the weight, *w*, given by the stakeholder for that criterion, *m*. MCDA produces a ranked list of alternatives for each individual country. While the model will indicate the preferred alternative given available information under existing circumstances, it is highly likely that *future* economic, environmental, political, and social needs will shift due to a variety of factors. As such, it is crucial to understand how easily preference may shift in this model from one alternative to another. Sensitivity analysis is used to relax certain conditions and incrementally shift criteria weights (holding all other element constant) to identify the threshold at which the preferred alternative changes. If a significant change in criteria weights or score inputs required in order to change the preferred alternative, the alternative is said to be insensitive to the parameters. If optimality is perceived to shift easily or often, these results are determined to be sensitive to the criteria considered, and policymakers will need to take into account which alternatives may offer the best utility over time instead of simply the currently preferred alternative.

### Case study: predicting hydraulic fracturing policy with archetypal examples

This case study is designed to demonstrate how MCDA may be used to predict future state behavior regarding the regulation of unconventional drilling activities. Five archetypal countries were created to represent different combinations of social and economic pressures (Table 1). Specifically, each nation is modeled with unique environmental, economic, social, and political factors that policymakers must consider as they decide how to regulate hydraulic fracturing. All are considered to have some volume of natural gas that could be acquired only through the process of hydraulic fracturing.

**Table 1:** **Characteristics of archetypal countries**

**Country archetype**	**Political structure**	**Economic potential**	**Environmental situation**	**Social trust in government**
1 Developed democratic	Capitalist democratic	High energy demand; significant gas reserves	Some environmental protection	Moderate
2 Former communist social democratic	Socialist democratic	High energy demand; moderate gas reserves	Limited environmental protection in favor of industrialization	Limited
3 Developing communist	Communist – centrally planned	Growing energy demand; significant gas reserves	Limited environmental protection in favor of industrialization	Limited
4 Developing third-world social state	Socialist democratic	Energy diversification; some natural gas potential	Substantial environmental protection	Moderate
5 Developed social democratic	Socialist democratic	High energy demand; limited tight gas reserves	Substantial environmental protection	High

In this limited case study, it is already apparent that the factors noted in Table 1 are highly varied and difficult to collectively assess for an individual country. Using MCDA, this information is quantified to for use as scores that can then be aggregated with sub-criteria weights. For now, the MCDA predictor is focused upon the four policy alternatives discussed above, although more can be integrated for additional analysis. The DECERNS software tool was used to evaluate the model using the multi-attribute value theory method (Figure 1).

## Abbreviation

MCDA: Multi-criteria decision analysis
